# Out-Patients at the Brompton Hospital

**Published:** 1889-08-31

**Authors:** 


### The Hospital Workshop.

### VIII.—OUT-PATIENTS AT THE BROMPTON HOSPITAL FOR CONSUMPTION AND DISEASES OF THE CHEST.

(BY OUR SPECIAL COMMISSIONER.)

This charity was founded in 1841. It stands in the Fulham-road, and since the opening of the new building in 1846, no less than 374,079 out-patients have come under treatment. On the opposite side of the road from the main building, but connected with it by a subway, lies an extensive wing, where the out-patient department is situated. Its rooms are handsome, well lighted, and spacious. The ceilings are lofty, 19½ft. high, and ventilation is carried on by pipes leading into extracting shafts heated with steam. These arrangements are made in view of modern theories, which trace the spread of consumption to the now famous "tubercle bacillus." It is now tolerably certain that some of the ways in which the disease is communicated are being discovered—for example, through the milk and flesh of tubercular cows. Striking experiments have lately been made by a Dr. Cornet, who replaced in an old feather bed a hundred feathers charged with the bacillus and carefully marked. He then had the bed disinfected no less than six times by a professional disinfector. Guinea-pigs were inoculated from five of the infected feathers on each occasion, and tuberculosis resulted in every case.

The responsibility of examining into the means of applicants is thrown upon subscribers, by whose letters patients are admitted. In all doubtful cases verbal enquiries are made by a clerk, and if the answers be not satisfactory, references are asked for to clergymen or other trustworthy people. All treatment is free. In the waiting-room are placed boxes to which donations are invited, and these produce on an average from £8 to £10 a quarter. Of the patients, some few come from the very poor, but on the whole they are of a fairly well-to-do class. Many sad histories are told in answer to the question "Why are you here? Are you not able to pay for medical advice?" For instance, a well-dressed girl replied, "Twelve months ago I should have been perfectly well able to do so. My father was an army man, but is now dead, and I am in a mantle-room in Oxford-street." Another said, "My husband was a clerk in the City, in a very good position, but since he has been obliged to give up his employment I have kept the family by teaching music." These are cases—if proved—that no one, however strong an objector to indiscriminate charity, could object to relieving. Moreover, many patients attend here for long periods, even for years together, and are supplied with expensive medicines such as cod liver oil and quinine. Some idea of the amount of oil given away may be gathered from the fact that it is ordered in quantities of 1,500 gallons at a time.

On an average there are about 230 out-patients daily. They are drawn from London and various parts of the country. As the secretary, Mr. Dobbin, remarked, "It would be difficult to say where they do not come from. Many occupations are represented: masons, bricklayers, cabmen, policemen, gentlemen's servants, dressmakers, female operatives, machinists, domestic servants. A number are entered from the engineers' workshops at Swindon. Many hail from Luton, where they are engaged in the straw-plait making. Although in this case the disease cannot be directly traced to the occupation, yet, as one of the physicians remarked, vegetable dust is very irritant to the lungs. This is shown by the frequency of bronchitis, asthma, and consumption among the cotton operatives of the North of England, and here at Brompton by the same affections among the chaff-cutters. Dust sets up inflammations of various kinds in the air-passages and opens the door to the tubercle bacilli, for it is a well-known fact that those organisms fasten chiefly, if not altogether, on weak or damaged tissues. Were it not for this resistance by the healthy, consumption would spread like wildfire, since every patient so affected would become a baleful centre of infection. The "dust diseases" as they are called, affect millers, bakers, coal-miners, stone-masons, and file-cutters. In the old days of dry grinding, the Sheffield steel-grinders suffered terribly, but the use of wet stones is now compelled by Act of Parliament.

All patients, upon arrival, are given a numbered ticket, which settles priority of entrance to the consulting-room. Only those who are obviously very ill, or those who come from long distances, are allowed to break this order. New cases are first seen by a clerk, who enters particulars of age, residence, and so on. He also enquires as to means of applicant, and looks up references to former treatment.

The title of this institution shows that besides consumption it treats other diseases of the chest. Accordingly, a large number of patients attend who are suffering from heart affections. "Chest," however, is a wide term. Dr. Milner Fothergill somewhere points out that a dyspeptic Cockney always complains of pain in the "chest," which with him means a region reaching far below the midriff. In this way many people with abdominal trouble present themselves at Brompton, and "dyspepsia" is by no means an uncommon entry. At the root of another large class of cases is anæmia, or "want of blood," from the fact of palpitation and shortness of breath being among its chief symptoms. So that a glance into the waiting-room does not reveal such a wasted and melancholy set of people as one might expect to see, and, indeed, many look quite healthy and robust. One must not judge by appearances, however, for sometimes the sufferer looks well even when examination reveals extensive lung mischief. The advanced cases of consumption are comparatively few and far between, but can hardly be mistaken. The face is ghastly white and marked with hard lines, especially about the mouth; the eyes are hollow and sunken, the breathing is quick, the back bowed, the knees bent, and the patient leans against the wall, or takes hold of a table or chair. The temperament is always hopeful, and the physician does not hesitate to tell the consumptive how bad he is; the news does not depress nor shock him as might happen in other diseases. A nurse attends in the waiting-hall with soup, milk, coffee, and eatables, which are sold at moderate prices. One old woman, after having had three penny basins of soup, was heard to declare that she had not enjoyed so good a dinner for many weeks.

Following our usual plan, let us visit one of the rooms where consultations are going on. An introduction to Dr. Hector Mackenzie, assistant physician, obtained the privilege of seeing a series of instructive cases, of which a few notes may be interesting to readers of The Hospital.

A young man of weedy, town-bred type. He is thin and undersized. His forehead is high, broad, and bulbous, in sharp contrast with the small features and slender under jaw. His neck is bent, his dark eyes wistful, and mien attentive. As a rule, very little of what is going on escapes your consumptive. This young man is twenty-four years of age, clerk at a railway bookstall on the underground railway. Three years ago he had spitting of blood. Lately he has lost a good deal of weight, and has been much troubled with cough and "night sweats." All these are symptoms of importance in phthisis, a name which, by the way, means "wasting." Another great point is the family history. In the present instance the father died of consumption, and his brothers and sisters at an early age. Examination of the chest shows the lung to be much affected. The poor fellow's outlook is a bad one. He is advised, if possible, to change his employment, as the atmosphere of the underground railway cannot fail to be injurious. He says he will make the attempt, but does not seem at all hopeful about being able to do so.

The loss of flesh is a marked feature of many grave diseases. It is sometimes evident to the trained eye of the physician at a glance from the looseness of the patient's clothes. But in other cases it is advisable to get the exactitude of a weighing machine. One man told us he had tried three machines, all of which differed. One of them he mounted a second time, and pointed out to the proprietor that he could hardly have grown two pounds lighter in as many minutes. A tall, thin, black-bearded man leaning against the wall yonder has lost 10lb. weight in the last eight weeks, and 35lb. in three years. During that time he has twice been an inmate of the wards at Brompton, and has also been sent to Bournemouth. Dr. Mackenzie tells him to get another letter and he will send him to the seaside again.

Here is a girl whose fine dark eyes fail to redeem the painful whiteness of her sharp-featured face. She tells the kind of tale one might expect from her appearance. No appetite; very short of breath; always feel tired; coughs a good deal. Examination shows bronchitis, but the chief mischief is poverty of blood. Chronic bronchitis is a common complaint here, chiefly met with in persons of advanced age. This cherry-cheeked school teacher from the country is another case of anæmia, in spite of her colour. She complains of pain in the chest and had suffered much from dyspepsia for three years past; indeed, she says her diet during that period has been almost entirely milk and lime-water. The "stethoscope," or tube through which the physician listens to sounds within the chest, reveals what are called "anæmic murmurs" over the big veins that empty into the heart. They are called by the French "*bruits de diable*," and make a most peculiar noise, best likened, perhaps, to the whistle of a humming-top.

The throat is often examined here, for the consumption process in its later stages attacks the vocal chords as well as the food passages. The eye is also affected by tubercle, but such cases generally find their way to ophthalmic hospitals.

Dispensing is quickly done, and everything is arranged with that end in view. Most of the medicines are made in the place. A very large amount of lozenges is dispensed—thirty-two hundredweight in the year — and they are wrapped in variously-coloured papers to prevent mistakes. The directions on labels are printed in good bold type. In short, the arrangements of the whole department show care and forethought in every detail, but to do the courteous dispenser anything like justice would require a paper by itself.

